# Meta-Analysis of Genetic Programs between Idiopathic Pulmonary Fibrosis and Sarcoidosis

**DOI:** 10.1371/journal.pone.0071059

**Published:** 2013-08-14

**Authors:** Dong Leng, Caijuan Huan, Ting Xie, Jiurong Liang, Jun Wang, Huaping Dai, Chen Wang, Dianhua Jiang

**Affiliations:** 1 Clinical Laboratory, Beijing Chao-Yang Hospital, Capital Medical University, Beijing, China; 2 Laboratory Research Center, Beijing Chao-Yang Hospital, Capital Medical University, Beijing, China; 3 Beijing Key Laboratory of Respiratory and Pulmonary Circulation Disorders, Beijing Institute of Respiratory Medicine, Beijing, China; 4 Division of Pulmonary, Allergy, and Critical Care Medicine, Department of Medicine, Duke University School of Medicine, Durham, North Carolina, United States of America; 5 Beijing Hospital of Ministry of Health, Beijing, China; 6 Cedars Sinai Medical Center Department of Medicine, Los Angeles, California, United States of America; Texas A & M, Division of Cardiology, United States of America

## Abstract

**Background:**

Idiopathic pulmonary fibrosis (IPF) and pulmonary sarcoidosis are typical interstitial lung diseases with unknown etiology that cause lethal lung damages. There are notable differences between these two pulmonary disorders, although they do share some similarities. Gene expression profiles have been reported independently, but differences on the transcriptional level between these two entities have not been investigated.

**Methods/Results:**

All expression data of lung tissue samples for IPF and sarcoidosis were from published datasets in the Gene Expression Omnibus (GEO) repository. After cross platform normalization, the merged sample data were grouped together and were subjected to statistical analysis for finding discriminate genes. Gene enrichments with their corresponding functions were analyzed by the online analysis engine “Database for Annotation, Visualization and Integrated Discovery” (DAVID) 6.7, and genes interactions and functional networks were further analyzed by STRING 9.0 and Cytoscape 3.0.0 Beta1. One hundred and thirty signature genes could potentially differentiate one disease state from another. Compared with normal lung tissue, tissue affected by IPF and sarcoidosis displayed similar signatures that concentrated on proliferation and differentiation. Distinctly expressed genes that could distinguish IPF from sarcoidosis are more enriched in processes of cilium biogenesis or degradation and regulating T cell activations. Key discriminative network modules involve aspects of bone morphogenetic protein receptor two (BMPR2) related and v-myb myeloblastosis viral oncogene (MYB) related proliferation.

**Conclusions:**

This study is the first attempt to examine the transcriptional regulation of IPF and sarcoidosis across different studies based on different working platforms. Groups of significant genes were found to clearly distinguish one condition from the other. While IPF and sarcoidosis share notable similarities in cell proliferation, differentiation and migration, remarkable differences between the diseases were found at the transcription level, suggesting that the two diseases are regulated by overlapping yet distinctive transcriptional networks.

## Background

Idiopathic pulmonary fibrosis (IPF), the most common type of idiopathic interstitial pneumonia, is a chronic, progressive, irreversible and lethal lung disease of unknown etiology [1], [2], [3]. The incidence of IPF is estimated at 7–10 cases per 100,000 per year with a presentation age of 50–70 years and no predilection by race or ethnicity [1], [4]. Previous studies have shown that the alveolar epithelial cells rather than the inflammatory cells play a vital role in the initiation of the fibrogenic events, and a variety of cytokines and growth factors expressed by injured or activated alveolar epithelial cells are key actors in the development of IPF [1], [5], [6].

Sarcoidosis, a multisystemic disorder, is defined as the manifestation of immune granulomas found in many organs. The incidence varies depending on age, sex, race and geographic factors, but the predominantly affected systems are the lungs and lymphatic system [7], [8], [9], [10]. Like IPF, the etiology of sarcoidosis remains unknown, and the prevailing hypothesis is that it is a chronic and exaggerated immunological response related to genetic susceptibility, specific infections or environmental factors [11], [12]. We reported that human leukocyte antigen polymorphisms may have a role in susceptibility and manifestation of sarcoidosis [13]. Cytokines like TNF-α, IL-12 and interferon gamma (IFN-γ) have been revealed to be involved in the formation of sarcoidosis [14], [15].

The extensive use of microarray technology has had a profound impact on characterizing the transcriptional changes of lung diseases. Microarray studies based on different platforms using different sample sources (lung tissue, isolated cells or blood) have provided a huge amount of information about lung disease processes, and have been successfully used in determining gene expression patterns or finding potential biomarkers [16]. Gene expression profiles based on microarray technique have been successfully applied to indicate the gene expression patterns of IPF and its potential biomarkers [2], [17], [18], [19], [20]. Gene expression analysis was also attempted to find potential pathogenic mediators of pulmonary sarcoidosis and to provide evidence that sarcoidosis causes intense immune responses like hypersensitivity pneumonitis and IPF [21], [22]. Meta-analysis of microarray data with the aim to identify significantly expressed genes and important signaling pathways across different platforms has been applied to cancer and gave high predictive models which are more validated than single-set analysis [23]. However, integrated comparison analysis has not been performed to compare the two typical interstitial lung disorders IPF and sarcoidosis.

This study was designed to identify genetic programs that differentially regulate IPF and sarcoidosis based on previously published microarray data from the Gene Expression Omnibus (GEO) datasets. We found similarities and diversities in gene expression between IPF and sarcoidosis. Our results also provided novel signature genes for pathogenic analysis and diagnostic or therapeutic purposes.

## Methods

### Study Datasets

Expression profile data related to either idiopathic pulmonary fibrosis or sarcoidosis were acquired from the GEO repository [24] (http://www.ncbi.nlm.nih.gov/projects/geo/). In this study, datasets of all human lung tissue were used for further analysis and, to make comparisons across platforms, datasets by commonly used platforms like Affymetrix and Agilent were retained.

### Expression Intensity Extracting and Probe Mapping

Selected datasets downloaded from the GEO repository in different forms contain different forms of expression measurements and probe annotation files. In particular, expression intensities of Affymetrix HGU133 Plus 2.0 were saved in.CEL files, and can be extracted by robust multi-array average (RMA) using software Affymetrix® Expression Console™ version 1.1.

Probes and their measurements of each expression profile from different platforms were all mapped to a common gene list as previously described [25]. Probes of different data were replaced by official gene symbols, and multiple expression measurements were collapsed by median value when one gene has reduplicative measurements [26], [27].

### Data Processing

All expression estimates were log_2_ transformed and then merged by cross platform normalization (xpn) which were performed using R 2.14.1 and Bioconductor package CONOR [27], [28]. Two expression data of different studies with the same common gene symbols were normalized and produced a new dataset, and then the newly produced data was renormalized with the next data. Finally, expression measurements of one study can be compared across all study populations within common genes.

### Statistical Analysis

#### Unpaired student’s t-tests

In order to find different expressed genes and make comparisons over normal, IPF and sarcoidosis lung tissues, we carried out unpaired student’s t-tests on the integrated super array data. Significantly expressed genes are defined as those with *P*<0.05, the same criterion for discovering differently expressed genes [2], [20], and higher statistical significances at *P*<0.01were used by Lockstone et al. [21] and at *P*<0.005 by Crouser et al. [22].

#### Unsupervised clustering analysis

Significantly expressed genes were analyzed by hierarchical clustering algorithms using the software Gene Cluster 3.0. Expression estimates were first adjusted by centering genes and arrays, then followed by two-way clustering (TWC) using the Euclidean distance similarity metric and the complete linkage clustering method, and clustering results were visualized by Java TreeView. Unsupervised TWC analysis can not only reveal the expression trend of genes but also isolate the outlier samples that possess unique expression features. We carried out our data mining by alternately using unpaired student’s t-tests and TWC until one circumstance can be differentiated from another completely.

#### Supervised classification

To get significantly expressed genes for each comparison, (comparisons between normal and IPF, between normal and sarcoidosis, and between IPF and sarcoidosis), the supervised learning method was carried out on the final unsupervised TWC clustering results using the software Significance Analysis of Microarrays (SAM) 4.0 (http://www-stat.stanford.edu/~tibs/SAM/) [30], which will produce more significant expressed genes with lower false discovery rate (FDR or the q-value). During classifications, the response type of two class unpaired was chosen, and arrays were median centered before analysis. The minimum fold change was set by two to get more obvious significantly expressed genes as far as possible. The top 130 significant genes with the highest SAM scores that equals to the T-statistic value (65 top significantly up-regulated genes and 65 top significantly down-regulated genes) were used as the signature for each comparison in our study. This was adopted as in Meltzer’s study, where151 genes (probesets in fact) which correspond to 136 unique genes that identified by Student’s t test were used to develop the IPF model, and 148 features from another dataset GSE10667 can mapped to features of the IPF model [2]. The threshold of 130 unique genes we use will capture main features for our super array data.

### Pathway and Network Analyses

Signature genes that can be used to discriminate each condition were subsequently submitted to the online software “Database for Annotation, Visualization and Integrated Discovery” (DAVID) 6.7 (http://david.abcc.ncifcrf.gov/) for inquiring functional annotations and gene enrichments [31], [32]. Also, significant expressed genes were mapped according to their direct or indirect interactions by the web-server STRING 9.0 (http://string.embl.de/) [33]. Complex gene networks related with different conditions were analyzed by Cytoscape 3.0.0 Beta1 (http://www.cytoscape.org/) [34].

## Results and Discussion

### Research Datasets and Integrated Data

**Figure 1:**
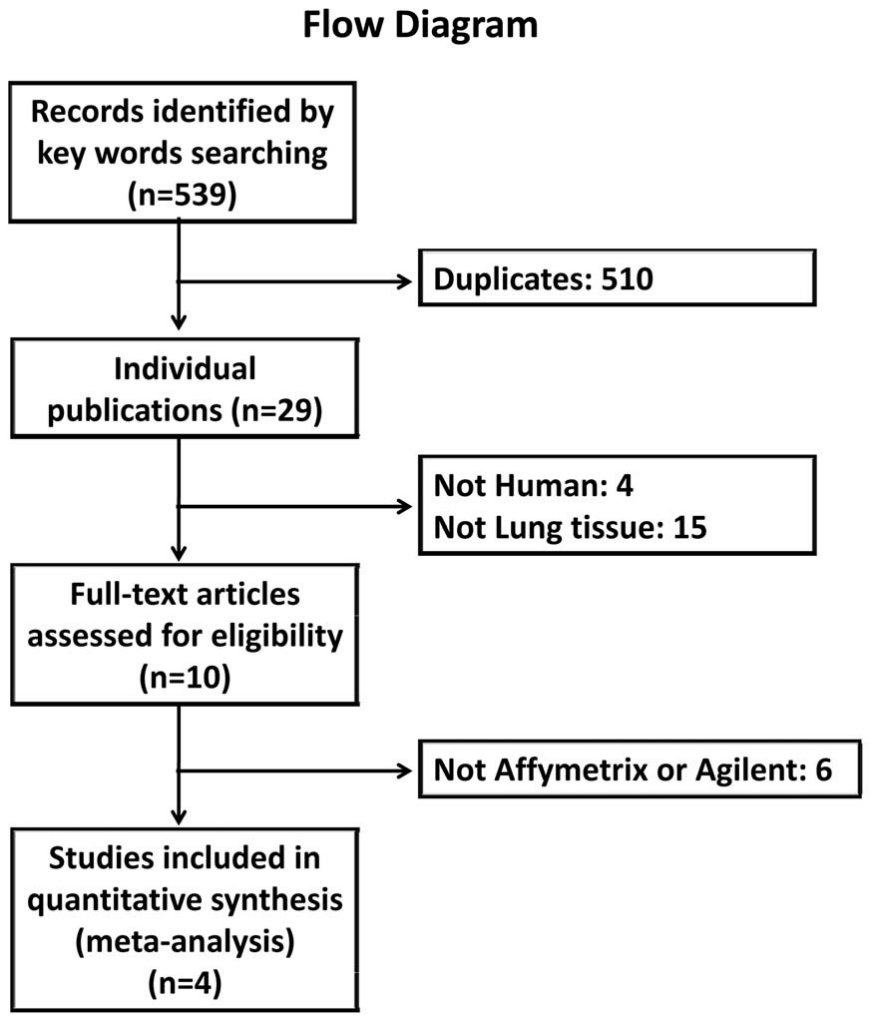
Flow diagram of this study. Idiopathic pulmonary fibrosis and sarcoidosis gene expression datasets were acquired through the Gene Expression Omnibus repository followed by exclusions of duplicates, non human lung tissues and non Affymetrix or Agilent datasets.

In this study, meta-analysis using lung tissue DNA microarray analyses crossing different studies by different platforms was performed in an attempt to make comparisons across lung tissue samples from normal, IPF and sarcoidosis, and to extract more validated genes together with relevant biological pathways for discriminating different conditions. By querying the key words “idiopathic pulmonary fibrosis” and “sarcoidosis”, five hundred and thirty-nine records are found in GEO database at present (218 IPF related and 321 sarcoidosis related records). After filtering datasets by organism of Homo sapiens and sample source of lung tissue, ten datasets remained (Table S1). In this study, four representative datasets are used for further analysis: GSE24206 (Affymetrix HGU133 Plus 2.0), GSE10667 (Agilent-014850 Whole Human Genome 4×44K Microarray), GSE19976 (Affymetrix HG 1.0 ST) and GSE16538 (Affymetrix HGU133 Plus 2.0). Four reports based on the above expression datasets with related patient information have been published [2], [20], [21], [22]. Flow diagram of this study is illustrated in Figure 1. And patient population for further analysis is summarized in Table 1.

**Table 1:** Patient characteristics for gene expression analysis.

Variable	Normal	IPF	Sarcoidosis
Average age, years	(51±10)^3^	(61±7)^1^, (67±11)^2^	(41±9)^3^, (52±17)^4^
Sex (male/female)	(3/3)^3^	(12/5)^1^, (25/6)^2^	(2/4)^3^, (8/7)^4^
Race (white/black/other)	(4/2/0)^3^		(4/2/0)^3^, (15/0/0)^4^
Smoking history (yes/no/unknown)	(5/1/0)^3^		(3/2/1)^3^, (0/15/0)^4^
Average FVC%		(65±19)^1^, (56±16)^2^	(85±13)^3^, (82±41)^4^
Average DLCO%		(53±35)^1^, (40±16)^2^	(71±16)^3^, (99±28)^4^

Abbreviations: FVC = forced vital capacity; DLCO = diffusing capacity of carbon monoxide;

1 Data from GSE24206;

2 Data from GSE10667;

3 Data from GSE16538;

4 Data from GSE19976.

To make comparable estimates for the four datasets, xpn method was carried out on the log_2_ transformed expression estimates of the selected four datasets. Cross-platform normalization (xpn) method based on a simple block-linear model is the procedure of measurements normalization of data from two or more studies. Gene expression estimates in each study are represented as specific matrix. Data normalizing procedure is an iterative clustering process until convergence to a local minimum of the squared Euclidean distance sum. Xpn is not gene-wise affine compared with other methods in the literature, which can successfully remove systematic differences between platforms while preserving biological information [27]. The log_2_ transformation is used to make our data more symmetric that will be helpful for plotting, and the transformation can also make the random variation more constant [29]. Probes of the four studies containing the corresponding expression intensities were mapped to the MAQC 12,091 common genes. Four datasets from four studies were normalized and have produced an integrated super array dataset. The super array data contains 96 lung tissue samples and 10,212 common genes in total (Table S2). All samples were assigned with new labels together with their GEO accession numbers. Measurements of the supper array were log_2_ transformed then xpn normalized. Here, 1,897 genes were filtered out as they were not shared by the four studies.

### Comparisons Across Normal, IPF and Sarcoidosis

**Figure 2:**
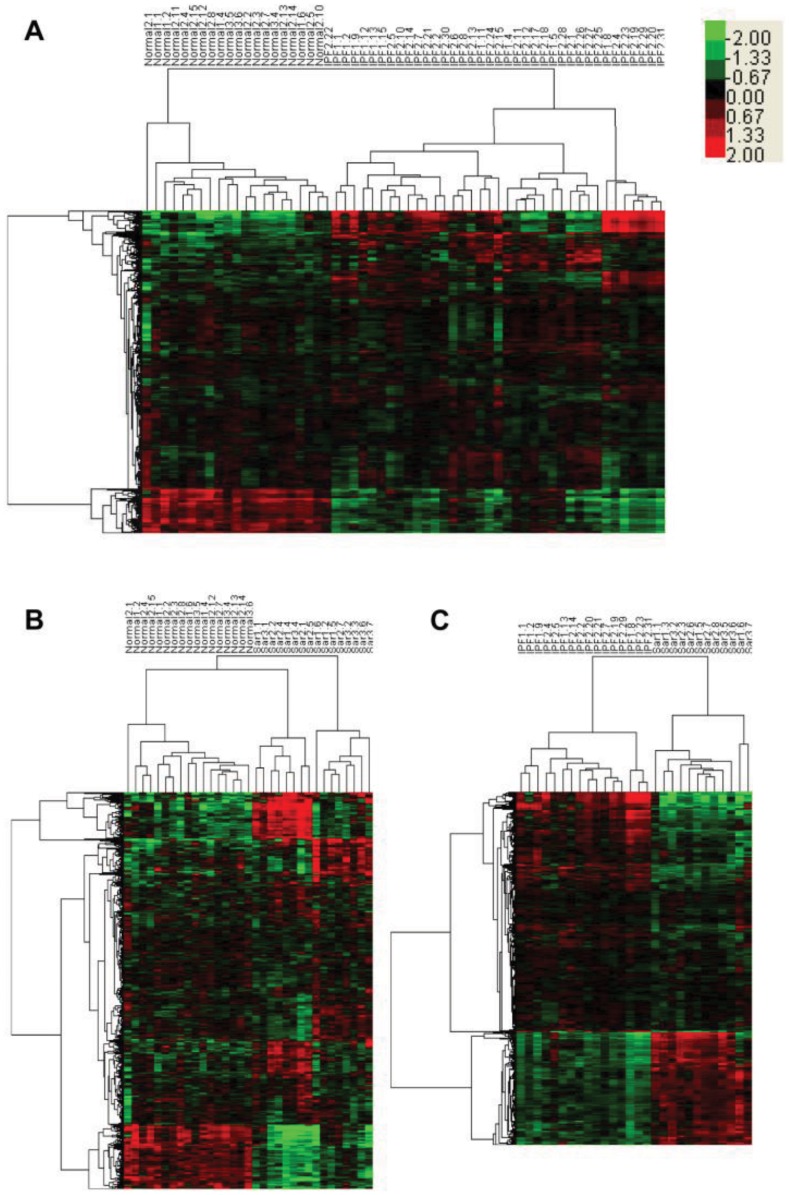
Comparison of samples from normal and IPF, normal and sarcoidosis, and IPF and sarcoidosis. Unsupervised TWC results for each comparison without outlier samples. (**A**) Unsupervised hierarchical clustering of samples of IPF (n = 38) and normal (n = 20) with 2,946 IPF up regulated and 466 IPF down regulated genes. (**B**) Unsupervised hierarchical clustering of samples of sarcoidosis (n = 16) and normal (n = 17) with 920 sarcoidosis up regulated and 176 sarcoidosis down regulated genes. (**C**) Unsupervised hierarchical clustering of samples of IPF (n = 16) and sarcoidosis (n = 12) with 2,051 IPF up regulated genes and 961 IPF down regulated IPF genes. Colorbar in the upper right corner shows the relative quantity of each gene, from light green to bright red corresponds to relatively lower to higher expressions. Abbreviations: Normal = normal lung tissue samples, IPF = idiopathic pulmonary fibrosis lung tissue samples, Sar = sarcoidosis lung tissue samples. Samples numbers correspond to those labeled in Table S2.

TWC was carried out on data with genes that are significantly expressed between two conditions (*P*<0.05). In order to get perfect clustering results, outliers with different clustering patterns were filtered out (see Table S3 for sample participations during clustering analysis, there are 27 normal, 48 IPF and 21 sarcoidosis tissue samples), and the appearance of outliers is the product of special gene expression patterns of special patients. When we compared normal and IPF samples, 20 normal and 38 IPF samples can make a perfect clustering pattern and 3,412 genes can be used to distinguish two conditions. Within the 3,412 genes, 2,946 genes and 466 genes showed up and down regulated expression patterns in IPF, respectively (Figure 2A). The same clustering analyses were also carried out on normal versus sarcoidosis and IPF versus sarcoidosis samples. 17 normal samples and 16 sarcoidosis samples can finally make a perfect clustering result (Figure 2B) and that produced 1,096 distinct genes (for sarcoidosis to normal samples, there are 920 up regulated and 176 down regulated genes). IPF and sarcoidosis can be discriminated by 3,018 genes (there are 2,057 up regulated and 961 down regulated genes when sarcoidosis is compared with IPF), and 16 IPF versus 12 sarcoidosis can make a perfect clustering result (Figure 2C). Potential valuable gene expression information could be lost from those filtered outlier samples. For comparison of normal vs. IPF, 17 samples/202 genes out of 75 samples/10,212 genes were removed. And for normal vs. sarcoidosis, 15 samples/284 genes out of 48 samples/10,212 genes were removed. Specially, for IPF vs. sarcoidosis, in 69 samples/10,212 genes, 41 samples were removed whereas significant expressed genes increases from 37 to 3,018. Outlier samples were removed to increase uniformity of datasets and significant expressed genes obtained from such uniform datasets could represent more typical features for each comparison. Differentially regulated genes for each comparison together with fold changes and p-values are listed in Table S4. No gender preference was found in our results, but some significant expressed genes were found to be related with previously published arrays. For instance, Genes MMP1, MMP7, AGER and COL1A2 were reported to be distinguished expressed from normal in IPF [20], and MMP12 was also mentioned to be different from normal in sarcoidosis [21], our findings for those genes have the same gene expression pattern related with those published data.

### Classification Produced Signature Genes

**Figure 3:**
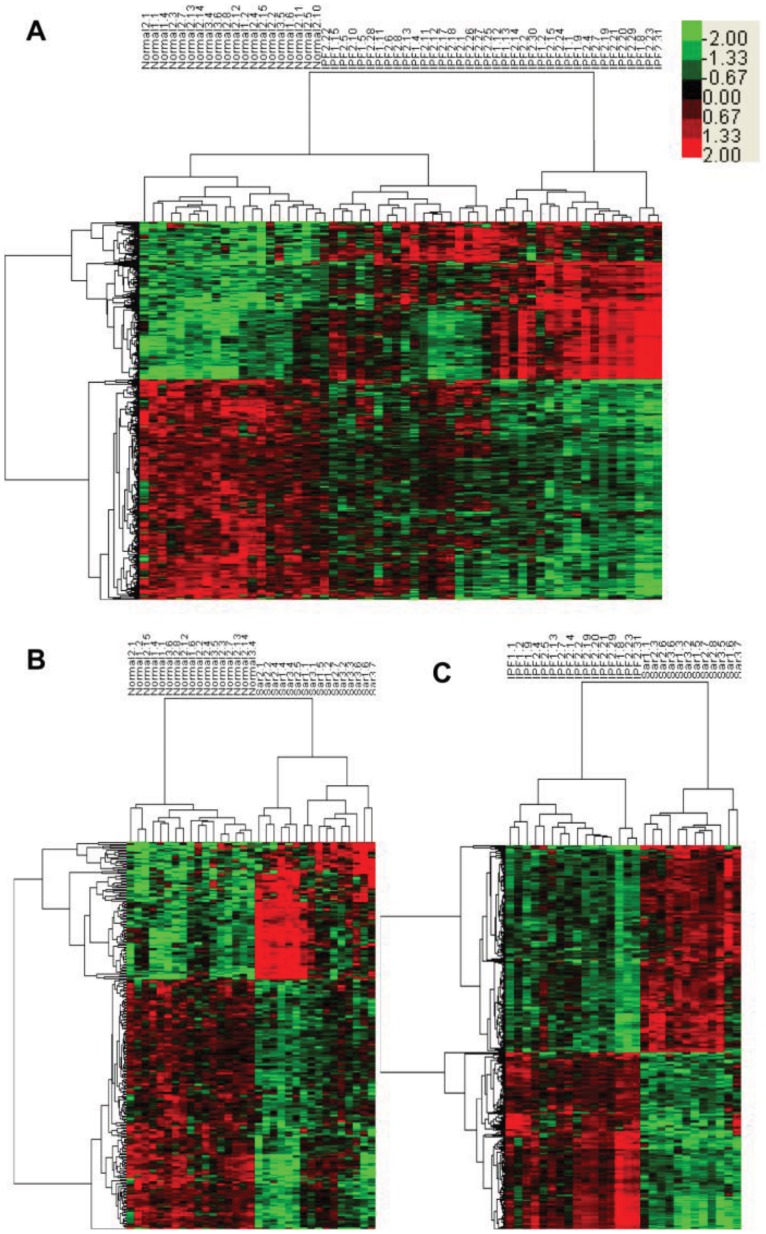
Significant expressed genes from normal vs. IPF, normal vs. sarcoidosis and IPF vs. sarcoidosis comparisons. Supervised classification results for each comparison using SAM based on unsupervised TWC results. (**A**) Hierarchical clustering of samples of IPF (n = 38) and normal (n = 20) with 197 IPF up regulated and 275 IPF down regulated significant expressed genes. (**B**) Hierarchical clustering of samples of sarcoidosis (n = 16) and normal (n = 17) with 96 sarcoidosis up regulated and 174 sarcoidosis down regulated significant expressed genes. (**C**) Hierarchical clustering of samples of IPF (n = 16) and sarcoidosis (n = 12) with 382 IPF up regulated and 326 IPF down regulated significant expressed genes. Colorbar in the upper right corner shows the relative quantity of each gene.

To further analyze the significance of genes that can differentiate one condition from another, the supervised learning method SAM was carried out based on the unsupervised TWC results, and that produced three groups of significant expressed genes for normal vs. IPF, normal vs. sarcoidosis, and IPF vs. sarcoidosis comparisons. IPF samples have 472 significantly expressed genes that could be used to distinguish from normal samples, which include 197 up regulated and 275 down regulated genes (Figure 3A). Sarcoidosis samples have 270 significantly expressed genes (96 up regulated and 174 down regulated genes) that can be used to differentiate from normal samples (Figure 3B). IPF can also be separated from sarcoidosis by 708 significant expressed genes which include 326 up regulated and 382 down regulated genes (Figure 3C). All signature genes for all comparisons are summarized in Table S5.

**Figure 4:**
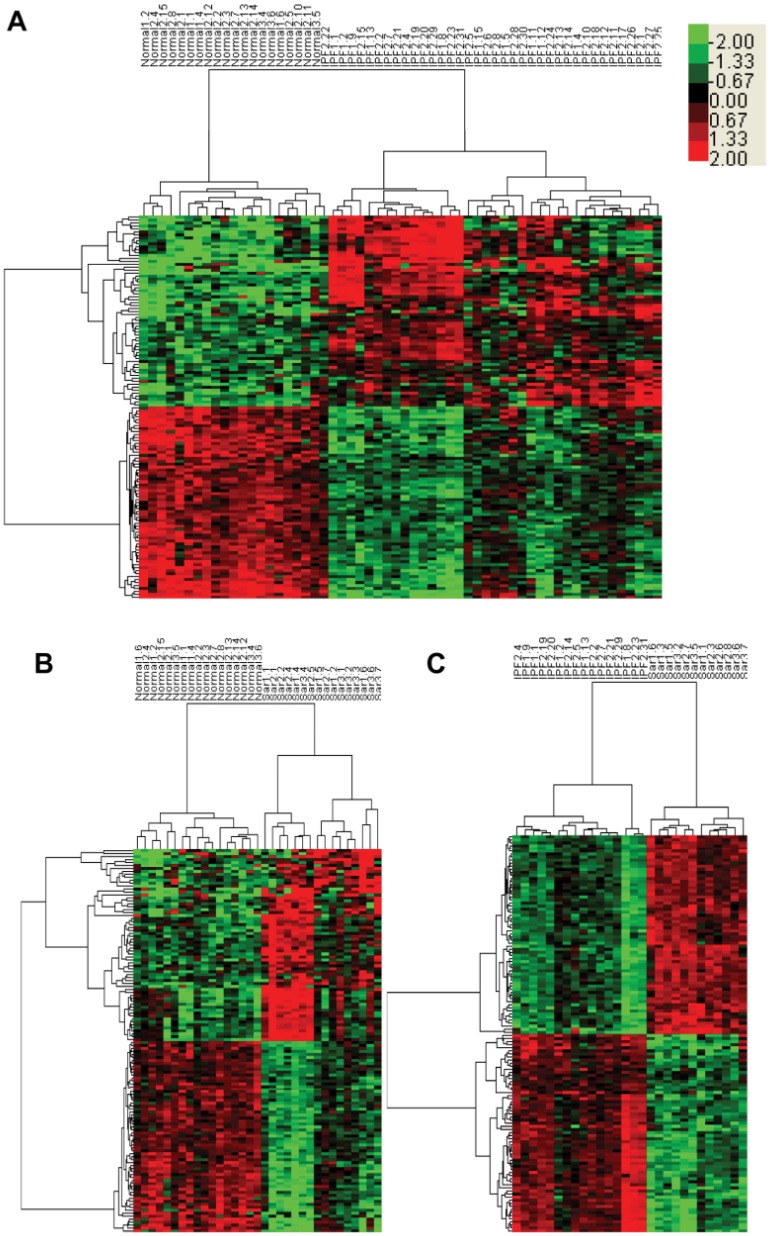
Signature genes from normal vs. IPF, normal vs. sarcoidosis and IPF vs. sarcoidosis comparisons. Top 65 up regulated and top 65 down regulated significant expressed genes extracted from SAM based on unsupervised TWC results. (**A**) Hierarchical clustering of samples of IPF (n = 38) and normal (n = 20) with top 65 IPF up regulated and top 65 IPF down regulated significant expressed genes. (**B**) Hierarchical clustering of samples of sarcoidosis (n = 16) and normal (n = 17) with top 65 sarcoidosis up regulated and top 65 sarcoidosis down regulated significant expressed genes. (**C**) Hierarchical clustering of samples of IPF (n = 16) and sarcoidosis (n = 12) with top 65 IPF up regulated and top 65 IPF down regulated significant expressed genes. Colorbar in the upper right corner shows the relative quantity of each gene.

The top 130 genes with highest SAM scores (top 65 up-regulated genes and top 65 down-regulated genes) in each comparison can be used as signature genes to separate the sample from the opposite condition more clearly (Figure 4). In both Figure 4A and 4B, we could find that in the disorder condition (either IPF or sarcoidosis), there are two clusters showing stronger or relatively weaker gene expression patterns. Stronger clusters do not show any patient or disease state preferences. IPF and sarcoidosis samples that possess stronger distinct signature gene expressions are randomly derived from different samples under different status. Signature genes are listed in Table S6. Distinct signature genes that could differentiate IPF or sarcoidosis from normal samples show strong similarities, 34 out of 65 up-regulated top signature genes and 33 out of 65 down-regulated top signature genes are shared by IPF versus normal and sarcoidosis versus normal comparisons, and those signature genes are either co-up regulated or co-down regulated. No cross regulated signature genes exist for comparison although signature genes can be found when directly comparing IPF with sarcoidosis. Signature gene expression patterns indicate that IPF and sarcoidosis do possess similarities which are not patient correlated.

### Gene Functional and Interactional Analyses

To illustrate the gene network of biological systems for each circumstance, signature genes were submitted to the online analysis software DAVID for gene enrichment and functional analysis. Gene-gene interactions were further retrieved and displayed for validating gene behaviors using STRING software.

**Figure 5:**
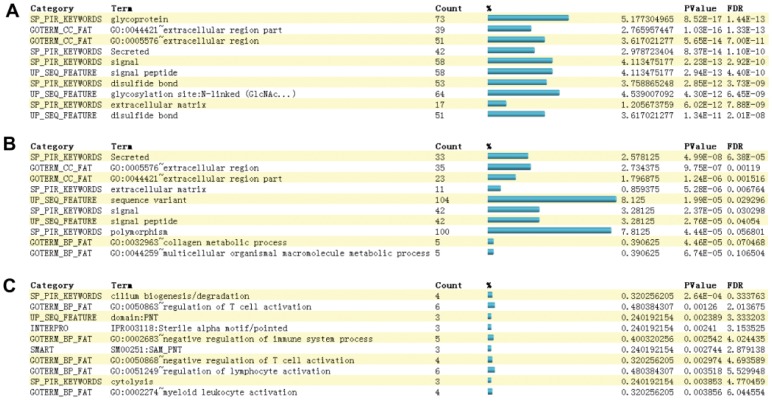
Functional annotation charts of distinct expressed genes. Panels A, B and C represent gene functional annotations of the top 10 of the 130 signature genes in comparisons of normal vs. IPF, normal vs. sarcoidosis and IPF vs. sarcoidosis. Terms are sorted according to the modified Fisher exact P-Values which test whether the categorization of a given gene list is more than random chance. The smaller, the more confident of the classification is. Genes and their percentages in each term were calculated accordingly.

In comparison with normal lung samples, IPF may have more glycoprotein-, signal-, disulfide bond-, and extracellular region-related features according to the gene enrichment (*P*<0.001) (Figure 5A). Similarly, sarcoidosis also has many extracellular region-, signal-, glycoprotein- and disulfide bond-related features (*P*<0.001) (Figure 5B). Therefore, IPF and sarcoidosis are similar disorders due to extracellular biological pathways. Most significantly expressed discriminative genes are more related to extracellular biological pathways. This confirmed the previous viewpoint that IPF and sarcoidosis possess characteristics of extracellular matrix accumulation in lung tissue [1], [12], [22], [35], and in some extent confirm that these disorders share some morphologic similarities [36].

However, they are different in their most enriched pathways. For IPF, the strongest term is “glycoprotein”, but for sarcoidosis, it is “secreted”. Glycoprotein is a term related to cell-cell interactions, but the term secreted is related to chemical compounds releasing or oozing processes. Comparison of IPF with sarcoidosis shows stronger gene enrichment in cilium biogenesis/degradation (*P*<0.001) and T cell activation (*P*<0.01) (Figure 5C). This may confirm that cell-cell interaction, for instance the activation of alveolar epithelial cells rather than inflammatory cells is the main pathogenic event for IPF but not for sarcoidosis, which could probably in turn confirm that fibrotic sarcoidosis is more similar to hypersensitivity pneumonitis than IPF [21].

**Figure 6:**
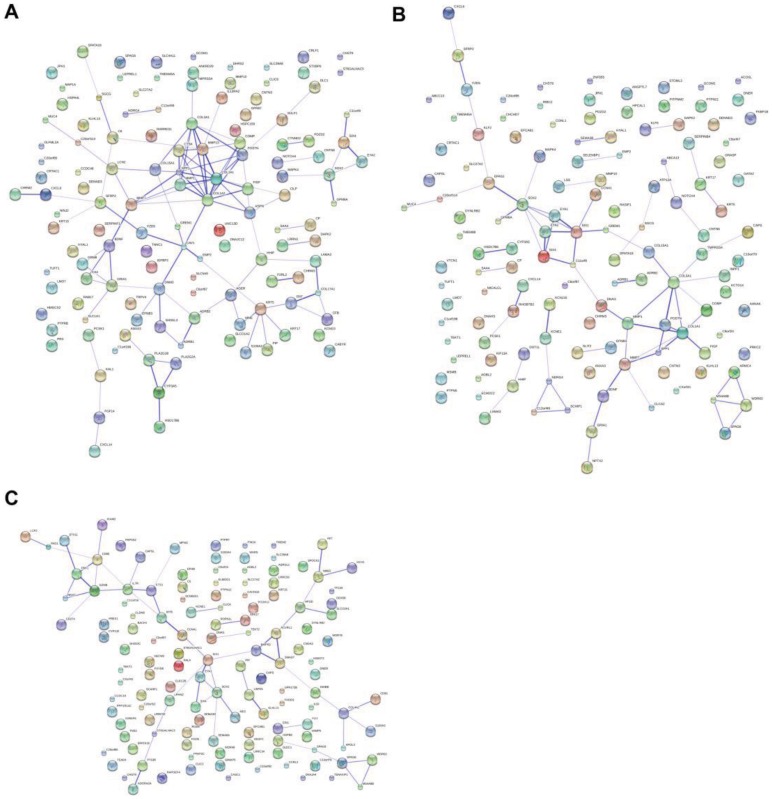
Interactions of major distinct expressed genes. Panels A, B and C show gene to gene interactive net work of the top 130 signature genes in comparisons of normal vs. IPF, normal vs. sarcoidosis and IPF vs. sarcoidosis. The darker strength of lines indicates stronger interactions between different genes.

For comparison between normal and IPF, significantly expressed genes strongly enriched in “glycoprotein” process show strong interactions on the protein level (Figure 6A). Eleven signature genes ASPN, POSTN, MMP1, MMP13, CTSK, COL1A1, COL3A1, COMP, FIGF, SPP1, and COL15A1 are the main interactive skeletons. From these interactive skeletons gene cluster, FIGF expression is down regulated, and the rest ten genes are up regulated. These interactive signature genes are mainly related with extracellular matrix and collagen changes. For instance, ASPN, a cartilage extracellular protein, POSTN, a periostin, and SPP1, a secreted phosphoprotein, are genes that could affect extracellular changes, especially ASPN, which inhibits the expression of transforming growth factor beta 1 (TGF β-1) and also induces collagen mineralization by binding collagen and calcium [37]. Matrix metalloproteinase family proteins MMP1 and MMP13 together with a noncollagenous extracellular matrix protein COMP here are distinctive, and their reproduction and tissue modeling activities reflect IPF development characteristics surrounding cell matrixes. Similarly, the fibrillar collagens COL1A1, COL3A1 and COL15A1 are over expressed and may play important roles in collagen accumulation. Particularly, the lysosomal cysteine proteinase CTSK also presents distinctively, and previous investigations have found that it collaborates fibroblasts in tumor invasiveness [38], possibly suggesting that IPF may possess expansionary tumor-like properties. The distinctive down-regulated gene that shows interactions with other signature genes is the gene FIGF, also known as vascular endothelial growth factor D. This gene was found to be active in angiogenesis, lymphangiogenesis and endothelial cell growth [39]. Lower expression associated with other signature genes may indicate that IPF is repressed in those aspects.

Significantly expressed genes of sarcoidosis compared with normal that also show some protein to protein interactions (Figure 6B) include 10 genes: COL3A1, MMP7, POSTN, GREM1, MMP1, BDNF, COMP, FIGF, SPP1, and COL15A1. Genes BDNF and FIGF are down regulated, and the rest eight genes in this interactive skeletons cluster are up regulated. In forming collagens and extracellular matrix properties, sarcoidosis also has over expressions in POSTN, SSP1 and GREM1 (a bone morphogenic protein) that used for collagen mineralization, in MMPs family protein MMP1, MMP7 and the COMP used to break down and form extracellular matrix, and in collagen proteins COL3A1 and COL15A1, used for collagen accumulations. The gene FIGF, which induces vessel, lymph or endothelial cell growth, is also down-regulated, and a nerve growth factor BDNF also shows lower expression characteristic for sarcoidosis, suggesting that sarcoidosis may reduce stress responses like Alzheimer’s and Huntington diseases [40].

Surprisingly, within the above significantly expressed genes that show strong interactive relationships, seven genes POSTN, MMP1, COL3A1, COMP, SOO1, COL15A1 and FIGF show consistency by IPF and sarcoidosis. This suggests that extracellular matrix forming or degradation and the accumulation of collagen could be the key similar biological pathways shared by IPF and sarcoidosis based on our research. By comparing with IPF, sarcoidosis shows significantly up regulated expression of CD274, IL7R and PAG1, which show strong protein to protein interactions (Figure 6C). Down-regulated genes CCNA1, NME5, SPA17 and SPAG6 show interactive effects. By contrast with IPF, up regulation of the sarcoidosis signature genes on interactive skeletons CD274, IL7R and PAG1 are all associated with T cell activations, and down regulated genes CCNA1, NME5, SPA17 and SPAG6 that show interactive activities are all associated with cell division. Discriminative transcriptional changes between IPF and sarcoidosis could be mainly revealed by the above differentiated interactive skeletons. These differences reflect the diseases’ developmental differentiations: proliferation of IPF is much higher than that of sarcoidosis but T cell activation is lower. That suggests that the two diseases could have different pathogenesis in aspects of immune responses and proliferations.

### Transcription Network Analyses

**Figure 7:**
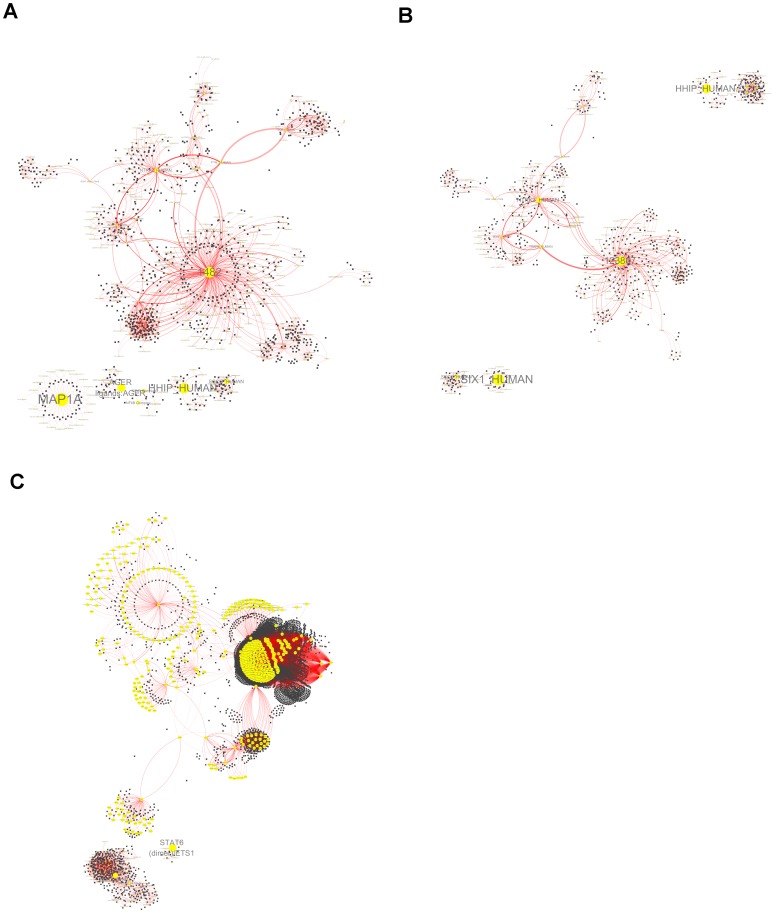
Functional network complexes and modules. Panel 6A–6C show functional network complexes for each set of signature genes in comparisons of normal vs. IPF, normal vs. sarcoidosis and IPF vs. sarcoidosis. Nodes represent functional networks and edges indicate network interactions.

Molecular interaction networks and functional modules were visualized by the open source bioinformatics software Cytoscape and the related plugin jActive Modules. Signature genes that could be used to distinct IPF from normal, to distinct sarcoidosis from normal, and to distinct IPF from sarcoidosis, were all submitted to Cytoscape, respectively, and that gave three gene network complexes as shown in Figure 7A–7C (the gene lists are in Table S7). Table S7 shows three network features for each comparison. The network features were extracted as modules for each complex. Network features of IPF and sarcoidosis are related with biological pathways of proliferation or differentiation. Discriminative network features that cause different performances are concerned with different signaling pathways. More reliable pathways concentrate on two aspects. For sarcoidosis, there is a mediator called BMPR2 (bone morphogenetic protein receptor, type II) that plays an important role and shows relatively higher expression compared with IPF, which has a relationship with TGF β-1. Recent studies have shown that TGF β superfamily collaborated with BMP cytokines can trigger proliferation, tissue regeneration, and angiogenesis, and BMPR2 was reported to be expressed higher on endothelial cell but lower on fibroblasts [41], [42]. The mediator MYB (v-myb myeloblastosis viral oncogene), which could trigger tumorigenesis, is overexpressed in IPF compared with that in sarcoidosis. It has been reported that MYB is a transcription gene that could sustain and enhance the inflammation process during breast cancer development [43], which potentially suggests that IPF possesses a higher risk of cancer development.

In-depth learning of critical genes that could draw interactive networks opened insight into pathogenesis. Critical gene networks provided descriptions of disease development in aspects of angiogenesis, neurotrophy and immune activations, which in turn reflect disease morphology. Our results suggest that IPF mainly results from epithelial injury/activation followed by inflammation in response to fibrosis. Excessive inflammation then leads to CD4^+^ T cell activation followed by formation of granulomas. Non self healing pulmonary sarcoidosis will develop to pulmonary fibrosis due to genetic variations [14], [44], [45], [46], [47]. However, limitations caused by data integration and clustering analysis could make us neglect some pathogenic factors.

Although a number of genes have been filtered out for analysis, we can pry out main genomic changes and provide information about pathogenic mechanism. Outlier samples that do not contribute to unsupervised clustering analysis are likely due to other risks of diseases. For instance, smoking has been confirmed to be an environmental factor of IPF [48], and evidence has also shown that sarcoidosis is affected by environmental exposure [49].

Although no complete signaling pathway has been described in this study, and no exact pathogenic mechanisms have been clearly illustrated for IPF and sarcoidosis, our study opened up new insights in genetic change analysis based on multiple databases, and our investigations may be more validated than single studies. Significantly expressed genes, especially those in critical modules used for discriminating different conditions, can be used to draw a regulatory map in disease development. Further studies with the aim of verifying gene expressions and investigating biological functions can at least in theory give a guide for new discoveries in pathogenic research.

### Conclusions

In this study, we performed meta-analysis with published datasets with IPF and sarcoidosis and found there were significant differences at the transcriptional level between them, while they did share some similarities. Compared with healthy lung tissue, IPF and sarcoidosis tissue are similar due to formations of extracellular matrixes and collagens. However, transcription networks containing significant expressed genes when comparing those two disorders further present discriminative signaling regulations by MYB and BMPR2, which suggest different prognoses. Furthermore, we believe that our research can promote illustrations of disorder mechanisms, and our method could be used as an essential tool for studying disorders on a transcriptional level.

## Supporting Information

Table S1 GEO Datasets Summary of IPF and Sarcoidosis. Summary of datasets that fit all criteria of what we used, filtered datasets were summarized by accession number, title, summary, organism, platform, total transcripts, total samples (n = 182), sample source, control (normal) sample number (n = 97), control (normal) sample sources, data format, data contributor and reference which the datasets were published.
(XLS)

Table S2 Integrated data. In order to get comparable data for further analysis, microarray estimates of selected datasets were merged by xpn method. The integrated data contains 96 lung tissue samples (27 normal, 48 IPF and 21 sarcoidosis lung tissues) of four different studies and 10,213 probes, known as common genes were included. For convenience, sample symbols were renamed according to the original studies.
(RAR)

Table S3 Sample participations during clustering analysis. To get perfect TWC results, outlier samples that overlap between two conditions were filtered out. When compare normal and IPF samples, 7 out of 27 normal samples and 10 out of 48 IPF samples were filtered out. In comparison between normal and sarcoidosis samples, 10 out of 27 normal samples and 5 out of 21 sarcoidosis samples were filtered out. In addition, 32 out of 48 IPF samples and 9 out of 21 sarcoidosis samples were filtered out. Key: positive signs = samples were used; negative signs = samples were filtered out; backslashes = samples were absent.
(XLS)

Table S4 Expression patterns for each comparison. Unsupervised clustering analysis (TWC method) was carried out to figure out general expression pattern for each comparison. Between normal and IPF, 20 out of 27 normal and 38 out of 48 IPF can be generally separated by 2,946 up regulated and 466 down regulated genes. Between normal and sarcoidosis, 17 out of 27 normal and 16 out of 21 sarcoidosis samples can be described by 920 up regulated and 176 down regulated genes. By comparing with sarcoidosis, IPF has 2,051 up regulated and 961 down regulated discriminative genes. Table S4 lists all discriminated genes for the above analysis, and the fold change and the corresponding p-value is calculated for each gene.
(XLS)

Table S5 Significant expressed genes for each comparison. Supervised classifications based on TWC results (SAM method) further extracted significant expressed genes that could be used to distinct one condition from another. Comparison of normal with IPF generated 197 up regulated and 275 down regulated significant expressed genes. Between normal and sarcoidosis, there are 96 up regulated and 174 down regulated significant expressed genes. To sarcoidosis, IPF has 382 up regulated and 826 down regulated significant expressed genes. Complete gene lists were summarized in Table S5.
(XLS)

Table S6 Signature genes. 130 significant expressed genes (65 up regulated and 65 down regulated) was selected as the key signatures for each comparisons, TWC analysis shown that they could be used to strongly discriminate normal and disease conditions, and for the comparison of normal versus IPF or the comparison of normal versus sarcoidosis, stronger or weaker sample cluster can well forms according to the expression measurements. The complete gene lists were in Table S6.
(XLS)

Table S7 Functional network complexes and modules. Signature genes of each comparison gave functional network complexes. The highly connected network regions are so called modules that could play vital role in diseases development. Table S7 describe the top two modules of each complex.
(XLS)

## References

[1] Pardo A, Selman M (2002) Idiopathic pulmonary fibrosis: new insights in its pathogenesis. Int J Biochem Cell Biol 34: 1534–1538. 12379275 10.1016/s1357-2725(02)00091-2

[2] Meltzer EB, Barry WT, D’Amico TA, Davis RD, Lin SS, et al (2011) Bayesian probit regression model for the diagnosis of pulmonary fibrosis: proof-of-principle. BMC Med Genomics 4: 70. 21974901 10.1186/1755-8794-4-70 PMC3199230

[3] King TE Jr, Pardo A, Selman M (2011) Idiopathic pulmonary fibrosis. Lancet 378: 1949–1961. 21719092 10.1016/S0140-6736(11)60052-4

[4] Crystal RG, Bitterman PB, Mossman B, Schwarz MI, Sheppard D, et al (2002) Future research directions in idiopathic pulmonary fibrosis: summary of a National Heart, Lung, and Blood Institute working group. Am J Respir Crit Care Med 166: 236–246. 12119236 10.1164/rccm.2201069

[5] Strieter RM, Keane MP (2004) Innate immunity dictates cytokine polarization relevant to the development of pulmonary fibrosis. J Clin Invest 114: 165–168. 15254582 10.1172/JCI22398 PMC449755

[6] Noble PW, Barkauskas CE, Jiang D (2012) Pulmonary fibrosis: patterns and perpetrators. J Clin Invest 122: 2756–2762. 22850886 10.1172/JCI60323 PMC3408732

[7] Statement on sarcoidosis. Joint Statement of the American Thoracic Society (ATS), the European Respiratory Society (ERS) and the World Association of Sarcoidosis and Other Granulomatous Disorders (WASOG) adopted by the ATS Board of Directors and by the ERS Executive Committee, February 1999. Am J Respir Crit Care Med 160: 736–755. 10430755 10.1164/ajrccm.160.2.ats4-99

[8] Moller DR (2007) Potential etiologic agents in sarcoidosis. Proc Am Thorac Soc 4: 465–468. 17684291 10.1513/pats.200608-155MS PMC2647598

[9] Nunes H, Bouvry D, Soler P, Valeyre D (2007) Sarcoidosis. Orphanet J Rare Dis 2: 46. 18021432 10.1186/1750-1172-2-46 PMC2169207

[10] Thomas KW, Hunninghake GW (2003) Sarcoidosis. JAMA 289: 3300–3303. 12824213 10.1001/jama.289.24.3300

[11] Wahlstrom J, Dengjel J, Persson B, Duyar H, Rammensee HG, et al (2007) Identification of HLA-DR-bound peptides presented by human bronchoalveolar lavage cells in sarcoidosis. J Clin Invest 117: 3576–3582. 17975675 10.1172/JCI32401 PMC2045606

[12] Baughman RP, Culver DA, Judson MA (2011) A concise review of pulmonary sarcoidosis. Am J Respir Crit Care Med 183: 573–581. 21037016 10.1164/rccm.201006-0865CI PMC3081278

[13] Zhou Y, Shen L, Zhang Y, Jiang D, Li H (2011) Human leukocyte antigen-A, -B, and -DRB1 alleles and sarcoidosis in Chinese Han subjects. Hum Immunol 72: 571–575. 21513758 10.1016/j.humimm.2011.03.020

[14] Grunewald J, Eklund A (2007) Role of CD4+ T cells in sarcoidosis. Proc Am Thorac Soc 4: 461–464. 17684290 10.1513/pats.200606-130MS PMC2647597

[15] Baughman RP, Lower EE, du Bois RM (2003) Sarcoidosis. Lancet 361: 1111–1118. 12672326 10.1016/S0140-6736(03)12888-7

[16] Tzouvelekis A, Patlakas G, Bouros D (2004) Application of microarray technology in pulmonary diseases. Respir Res 5: 26. 15585067 10.1186/1465-9921-5-26 PMC543572

[17] Kabuyama Y, Oshima K, Kitamura T, Homma M, Yamaki J, et al (2007) Involvement of selenoprotein P in the regulation of redox balance and myofibroblast viability in idiopathic pulmonary fibrosis. Genes Cells 12: 1235–1244. 17986007 10.1111/j.1365-2443.2007.01127.x

[18] Emblom-Callahan MC, Chhina MK, Shlobin OA, Ahmad S, Reese ES, et al (2010) Genomic phenotype of non-cultured pulmonary fibroblasts in idiopathic pulmonary fibrosis. Genomics 96: 134–145. 20451601 10.1016/j.ygeno.2010.04.005

[19] Boon K, Bailey NW, Yang J, Steel MP, Groshong S, et al (2009) Molecular phenotypes distinguish patients with relatively stable from progressive idiopathic pulmonary fibrosis (IPF). PLoS One 4: e5134. 19347046 10.1371/journal.pone.0005134 PMC2661376

[20] Konishi K, Gibson KF, Lindell KO, Richards TJ, Zhang Y, et al (2009) Gene expression profiles of acute exacerbations of idiopathic pulmonary fibrosis. Am J Respir Crit Care Med 180: 167–175. 19363140 10.1164/rccm.200810-1596OC PMC2714820

[21] Lockstone HE, Sanderson S, Kulakova N, Baban D, Leonard A, et al (2010) Gene set analysis of lung samples provides insight into pathogenesis of progressive, fibrotic pulmonary sarcoidosis. Am J Respir Crit Care Med 181: 1367–1375. 20194811 10.1164/rccm.200912-1855OC

[22] Crouser ED, Culver DA, Knox KS, Julian MW, Shao G, et al (2009) Gene expression profiling identifies MMP-12 and ADAMDEC1 as potential pathogenic mediators of pulmonary sarcoidosis. Am J Respir Crit Care Med 179: 929–938. 19218196 10.1164/rccm.200803-490OC PMC2684019

[23] Rhodes DR, Barrette TR, Rubin MA, Ghosh D, Chinnaiyan AM (2002) Meta-analysis of microarrays: interstudy validation of gene expression profiles reveals pathway dysregulation in prostate cancer. Cancer Res 62: 4427–4433. 12154050

[24] Barrett T, Edgar R (2006) Gene expression omnibus: microarray data storage, submission, retrieval, and analysis. Methods Enzymol 411: 352–369. 16939800 10.1016/S0076-6879(06)11019-8 PMC1619900

[25] Shi L, Reid LH, Jones WD, Shippy R, Warrington JA, et al (2006) The MicroArray Quality Control (MAQC) project shows inter- and intraplatform reproducibility of gene expression measurements. Nat Biotechnol 24: 1151–1161. 16964229 10.1038/nbt1239 PMC3272078

[26] Warnat P, Eils R, Brors B (2005) Cross-platform analysis of cancer microarray data improves gene expression based classification of phenotypes. BMC Bioinformatics 6: 265. 16271137 10.1186/1471-2105-6-265 PMC1312314

[27] Shabalin AA, Tjelmeland H, Fan C, Perou CM, Nobel AB (2008) Merging two gene-expression studies via cross-platform normalization. Bioinformatics 24(9): 1154–1160. 18325927 10.1093/bioinformatics/btn083

[28] Rudy J, Valafar F (2011) Empirical comparison of cross-platform normalization methods for gene expression data. BMC Bioinformatics 12: 467. 22151536 10.1186/1471-2105-12-467 PMC3314675

[29] Trevino V, Falciani F, Barrera-Saldana HA (2007) DNA microarrays: a powerful genomic tool for biomedical and clinical research. Mol Med 13: 527–541. 17660860 10.2119/2006-00107.Trevino PMC1933257

[30] Tusher VG, Tibshirani R, Chu G (2001) Significance analysis of microarrays applied to the ionizing radiation response. Proc Natl Acad Sci U S A 98: 5116–5121. 11309499 10.1073/pnas.091062498 PMC33173

[31] Huang da W, Sherman BT, Lempicki RA (2009) Systematic and integrative analysis of large gene lists using DAVID bioinformatics resources. Nat Protoc 4: 44–57. 19131956 10.1038/nprot.2008.211

[32] Huang da W, Sherman BT, Lempicki RA (2009) Bioinformatics enrichment tools: paths toward the comprehensive functional analysis of large gene lists. Nucleic Acids Res 37: 1–13. 19033363 10.1093/nar/gkn923 PMC2615629

[33] Snel B, Lehmann G, Bork P, Huynen MA (2000) STRING: a web-server to retrieve and display the repeatedly occurring neighbourhood of a gene. Nucleic Acids Res 28: 3442–3444. 10982861 10.1093/nar/28.18.3442 PMC110752

[34] Smoot ME, Ono K, Ruscheinski J, Wang PL, Ideker T (2011) Cytoscape 2.8: new features for data integration and network visualization. Bioinformatics 27: 431–432. 21149340 10.1093/bioinformatics/btq675 PMC3031041

[35] Rock JR, Hogan BLM (2011) Epithelial Progenitor Cells in Lung Development, Maintenance, Repair, and Disease. Annual Review of Cell and Developmental Biology, Vol 27 27: 493–512. 10.1146/annurev-cellbio-100109-104040 21639799

[36] Shigemitsu H, Azuma A (2011) Sarcoidosis and interstitial pulmonary fibrosis; two distinct disorders or two ends of the same spectrum. Curr Opin Pulm Med 17: 303–307. 21681100 10.1097/MCP.0b013e3283486d52

[37] Ikegawa S (2008) Expression, regulation and function of asporin, a susceptibility gene in common bone and joint diseases. Curr Med Chem 15: 724–728. 18336287 10.2174/092986708783885237

[38] Kleer CG, Bloushtain-Qimron N, Chen YH, Carrasco D, Hu M, et al (2008) Epithelial and stromal cathepsin K and CXCL14 expression in breast tumor progression. Clin Cancer Res 14: 5357–5367. 18765527 10.1158/1078-0432.CCR-08-0732 PMC2630242

[39] Ferrara N (2004) Vascular endothelial growth factor: basic science and clinical progress. Endocr Rev 25: 581–611. 15294883 10.1210/er.2003-0027

[40] Zuccato C, Cattaneo E (2007) Role of brain-derived neurotrophic factor in Huntington’s disease. Prog Neurobiol 81: 294–330. 17379385 10.1016/j.pneurobio.2007.01.003

[41] Ehrlich M, Gutman O, Knaus P, Henis YI (2012) Oligomeric interactions of TGF-beta and BMP receptors. FEBS Lett 586: 1885–1896. 22293501 10.1016/j.febslet.2012.01.040

[42] Gangopahyay A, Oran M, Bauer EM, Wertz JW, Comhair SA, et al (2011) Bone morphogenetic protein receptor II is a novel mediator of endothelial nitric-oxide synthase activation. J Biol Chem 286: 33134–33140. 21808054 10.1074/jbc.M111.274100 PMC3190885

[43] Bhattarai G, Lee YH, Lee NH, Yun JS, Hwang PH, et al (2011) c-myb mediates inflammatory reaction against oxidative stress in human breast cancer cell line, MCF-7. Cell Biochem Funct 29: 686–693. 21953443 10.1002/cbf.1808

[44] Antoniou KM, Tzouvelekis A, Alexandrakis MG, Sfiridaki K, Tsiligianni I, et al (2006) Different angiogenic activity in pulmonary sarcoidosis and idiopathic pulmonary fibrosis. Chest 130: 982–988. 17035428 10.1378/chest.130.4.982

[45] Dagnell C, Grunewald J, Kramar M, Haugom-Olsen H, Elmberger GP, et al (2010) Neurotrophins and neurotrophin receptors in pulmonary sarcoidosis - granulomas as a source of expression. Respir Res 11: 156. 21059230 10.1186/1465-9921-11-156 PMC2994818

[46] Heron M, van Moorsel CH, Grutters JC, Huizinga TW, van der Helm-van Mil AH, et al (2011) Genetic variation in GREM1 is a risk factor for fibrosis in pulmonary sarcoidosis. Tissue Antigens 77: 112–117. 21214523 10.1111/j.1399-0039.2010.01590.x

[47] Wallace WA, Howie SE (1999) Immunoreactive interleukin 4 and interferon-gamma expression by type II alveolar epithelial cells in interstitial lung disease. J Pathol 187: 475–480. 10398109 10.1002/(SICI)1096-9896(199903)187:4<475::AID-PATH268>3.0.CO;2-N

[48] Oh CK, Murray LA, Molfino NA (2012) Smoking and idiopathic pulmonary fibrosis. Pulm Med 2012: 808260. 22448328 10.1155/2012/808260 PMC3289849

[49] Culver DA, Newman LS, Kavuru MS (2007) Gene-environment interactions in sarcoidosis: challenge and opportunity. Clin Dermatol 25: 267–275. 17560304 10.1016/j.clindermatol.2007.03.005 PMC1920704

